# Allochthonous material originating from saprolite as a marker of termite activity in Ferralsols

**DOI:** 10.1038/s41598-022-21613-6

**Published:** 2022-10-13

**Authors:** Ary Bruand, Adriana Reatto, Éder de Souza Martins

**Affiliations:** 1 Institut des Sciences de la Terre d’Orléans (ISTO), UMR7327, UO-CNRS-BRGM, Observatoire des Sciences de l’Univers en région Centre, Université d’Orléans, 1A rue de la Férollerie, 45071 Orléans, Cedex 2 France; 2 Empresa Brasileira de Pesquisa Agropecuária (Embrapa), Secretaria de Pesquisa e Desenvolvimento, Parque Estação Biológica-PqEB S/n0, Brasília, DF Brazil; 3 Empresa Brasileira de Pesquisa Agropecuária (Embrapa Cerrados), Brasília, DF Brazil

**Keywords:** Element cycles, Environmental sciences

## Abstract

Ferralsols, which are estimated to cover 7.5 millions km^2^ worldwide, are deeply weathered red or yellow soils found in the humid tropics. They are considered as the end of a geochemical sequence of weathering and are dominated by low-activity clay and sesquioxides. Their physical properties are closely related to their strong submillimetric granular structure. We aimed to characterize the 2:1 clay minerals identified in many Ferralsols and to discuss them as a marker of soil-feeding termite activity in Ferralsols. We present results recorded with Brazilian Ferralsols developed under Cerrado native vegetation on a range of parent materials. It was found that the 2:1 minerals vary from weakly weathered muscovite to hydroxy-Al interlayered vermiculite, sometimes associated to a fine material with a chemical composition highly different from that of the groundmass of the surrounding submillimetric granular aggregates. Results show that both 2:1 minerals and the associated fine material have to be considered as allochthonous material originating from the saprolite and were brought to the Ferralsol by soil-feeding termite activity. This confirms the major role of termites in the properties of Ferralsols and raises questions about the possible consequences of land use change which usually deeply affects soil biodiversity.

## Introduction

Ferralsols are the deeply weathered red or yellow soils found in the humid tropics^1^. These soils show a poor horizonation with very diffuse limits between horizons. They are dominated by low-activity clay (mainly kaolinite) and a high sesquioxide content. They are considered as the end of a geochemical sequence of weathering^2–4^ and most show a weak macrostructure and strong submillimetric granular structure^5–9^. As indicated in the World Reference Base for soil resources^1^, Ferralsols often refer to Oxisols (United States of America), Latossolos (Brazil), Kandosols (Australia), Ferralitic soils (Russia), sols ferrallitiques or Ferrallisols (France) and Ferralítico, Alítíco and Ferrílitico (Cuba). Their worldwide extent is estimated at 7.5 million km^2^^1^. They are mainly developed on materials resulting from long and deep weathering of the rocks forming the continental shields of South America (Brazil) and Africa (Congo, Democratic Republic of the Congo, Angola, Guinea, southern Central Africa and Eastern Madagascar). Outside these regions, Ferralsols can be found in areas with easily weathered basic rocks and both a hot and wet climate such as Southern Asia^1^. In South America, Ferralsols are numerous and developed on a large range of parent materials in the Cerrado biome (2.1 million km^2^) where they represent 49% of the surface area^10^. Their possible degradation following native vegetation clearing and then agricultural development with its consequences on soil biodiversity is often questioned^11–14^.

Although the submillimetric granular structure has long been recognized in most Ferralsols as responsible for most of their physical fertility, its origin remains highly debated^7,15–18^. An increasing numbers of studies, however, favor a structure resulting from long term soil faunal activity, particularly soil-feeding termite activity^7,17–21^. This hypothesis has been mainly discussed for Brazilian Ferralsols where termites communities are abundant and where 2:1 clay minerals have been observed in the ferralic B horizons in contradiction with the geochemical evolution of the soils^7,20,22^. This is a major issue because if both the submillimetric granular structure and the presence of 2:1 clay minerals result from the action of soil-feeding termites under native vegetation, its clearing the vegetation to allow the development of intensive agriculture will cause a collapse of the soil biodiversity, as has been shown for other tropical native vegetations in Africa with a dramatic decrease in the number of soil-feeding termite species^22–24^. This has potentially dramatic consequences for the physical and chemical fertility of millions of hectares of Ferralsols that are physically fragile and chemically highly depleted due to their long and intense geochemical weathering^2–4^.

## Results and discussion

### Widespread presence of 2:1 clay minerals in ferralic B horizons

In this study, we looked for evidence of the presence of material originating from the saprolite in the ferralic B horizons of Brazilian Ferralsols where termites were suspected to bring saprolite material from the soil bottom^7,18,25^. These soils are good candidates for highlighting the presence of material originating from the saprolite which usually appears usually at several meters depth, as the mineralogical composition of their ferralic B horizons is very different from that of the underlying saprolite. Moreover, they host numerous soil-feeding termite communities^7,20,26–30^.

**Figure 1:**
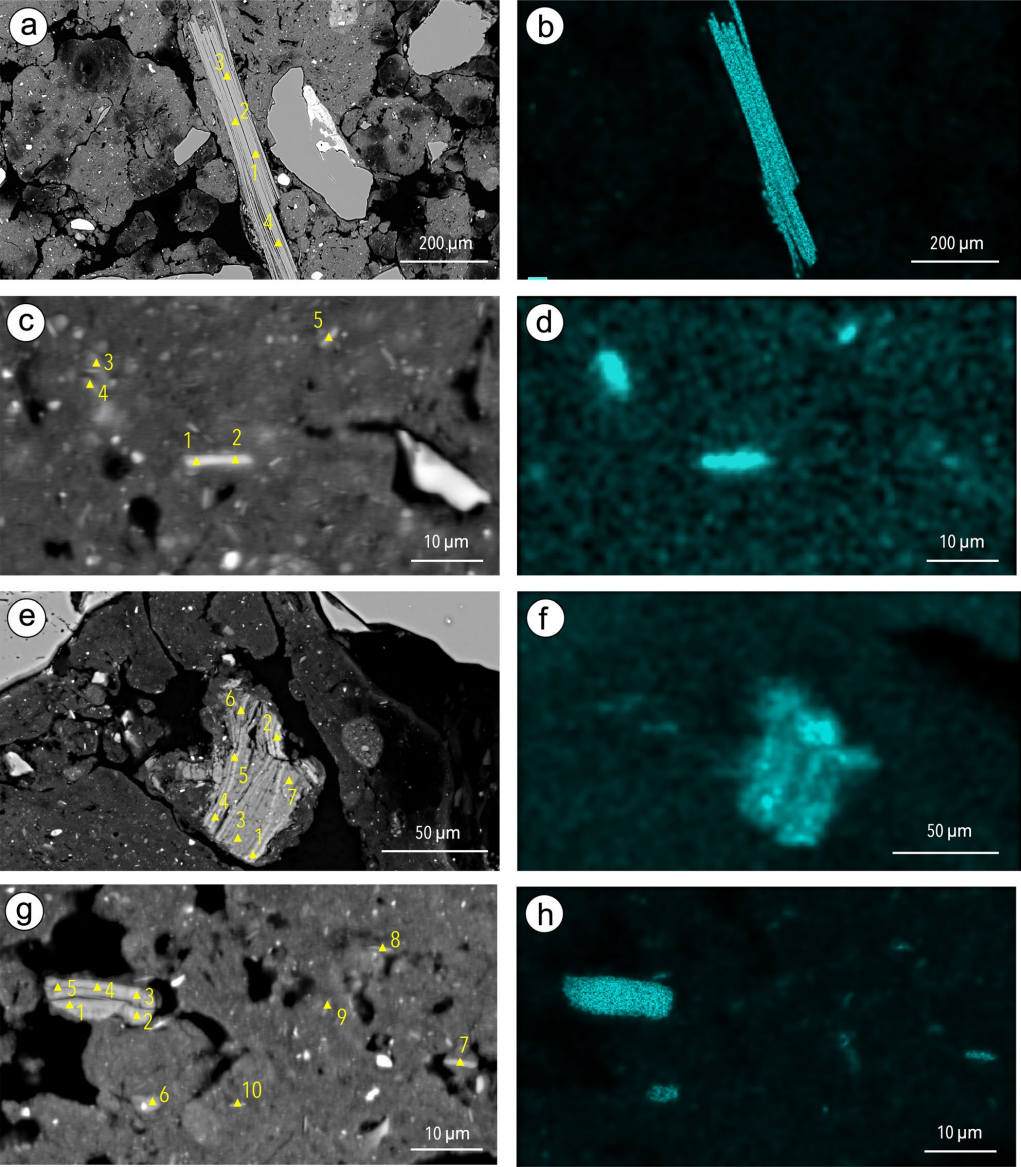
Elongated particles with varying sizes observed on the backscattered scanning images (BESI) of the cross sections of Ferralsols F1 (**a**), F2 (**c**), F3 (**e**) and F4 (**g**), location of the analyses (yellow triangles) performed by using energy dispersive spectrometry (EDS) (the numbers correspond to the analysis points in Supplementary Table 1), and K maps of the same areas using EDS for F1 (**b**), F2 (**d**), F3 (**f**) and F4 (**h**).

The backscattered electron scanning images (BESI) and maps of K content (Fig. 1), as well as the point chemical analyses carried out (Supplementary Table 1), showed that elongated particles with a K_2_O content ranging from 0.50 (Fig. 1e) to 10.87% (Fig. 1g) (Supplementary Table 1) were present in the ferralic B horizons of the four Ferralsols studied (“Methods”). According to earlier results, these elongated particles are 2:1 clay particles^18^. They ranged from a few microns in length belonging to the large clay- or fine silt-size fraction (Fig. 1c,d,g,h) to several hundred microns in length belonging to the fine sand-size fraction (Fig. 1a,b). Thus, they can be much larger than those evidenced earlier in Ferralsols^18^. Observation of the whole surface area of the polished cross section (“Methods”) showed that clay-size elongated particles (< 2 µm long) with a K_2_O content > 0.5% were highly numerous (i.e. > 50 particles for the 4.9 cm^2^ observed) in the Ferralsols F1, F2 and F3 and numerous (i.e. 10 to 50 particles for the 4.9 cm^2^ observed) in the Ferralsol F4 (Table 1). Fine silt-size particles (2–20 µm long) were highly numerous in F1 and F3 and numerous in F2 and F4 (Table 1). Similar large silt-size particles (20–50 µm long) were highly numerous only in F3, numerous in F1, poorly present (i.e. 1 to 10 particles for the 4.9 cm^2^ observed) in F2 and not observed in F4 (Table 1). Lastly, fine sand-size particles (50–500 µm long) were numerous in F1 and poorly present in F3 (Table 1). Thus, even if the elongated particles showing an internal structure of phyllosilicates varied in size, in abundance in the groundmass fabric of the submillimetric granular aggregates and in K_2_O content, they were present in the four ferralic B horizons studied and corresponded to the 2:1 clay minerals identified earlier using XR diffraction but without having the possibility to describe their morphology and to determine their chemical composition^29,31–37^.

**Table 1:** Length and number of elongated particles with K_2_O content > 0.5% which were observed on the BESI of the polished sections 4.9 cm^2^ in surface area (diameter of 2.5 cm).

Ferralsol	Length and number of the elongated particles			
	< 2 µm	2–20 µm	20–50 µm	50–500 µm
F1	> 50	> 50	10–50	10–50
F2	> 50	10–50	1–10	n.o
F3	> 50	> 50	> 50	1–10
F4	10–50	10–50	n.o	n.o

*n.o.* not observed.

The structural formula was computed for these 2:1 clay minerals on the basis of the structure of a 2:1 phyllosilicate. The results recorded for the analyses exhibiting the highest K_2_O content among the analyses performed (Fig. 1, Supplementary Table 1) and after attributing all Mg^2+^ to the octahedral sites showed that the number of occupied sites in the octahedral sheet ranged from 2.01 to 2.05 for the half unit cell in F1, F3 and F4 (Table 2). The electrical charge of the sheet for the half unit ranged from − 1.02 to − 1.03 and the electrical charge corresponding to the cations K^+^, Ca^2+^ and Na^+^ in the interlayer space from + 0.89 to + 0.97 (Table 2).

**Table 2:** Chemical composition of the half unit cell computed after location of the whole Mg^2+^ in the octahedral sites for the mean chemical composition resulting from the analyses of the particles varying in size shown in BESI of Fig. 1 and of the fine sand-size elongated particles shown in the BESI of Fig. 2.

Ferralsol	Location of the particle	Analyses taken into account	Chemical composition of the half unit cell	Number of octahedral cavities occupied	Electrical charge of the layer	Electrical charge of the cations in the interlayer space
F1	Figure 1a	Points 1 to 4	[Si_3.02_Al_0.98_]O_10_[Al_1.81_ Fe_0.10_Mg_0.07_Ti_0.03_](OH)_2_K_0.72_Ca_0.00_Na_0.25_	2.01	− 1.02	+ 0.97
F3	Figure 1e	Point 2	[Si_3.00_Al_1.00_]O_10_[Al_1.69_ Fe_0.25_Mg_0.07_Ti_0.04_](OH)_2_K_0.84_Ca_0.01_Na_0.06_	2.02	− 1.03	+ 0.92
F4	Figure 1g	Points 1 to 5	[Si_3.01_Al_0.99_]O_10_[Al_1.82_ Fe_0.16_Mg_0.05_Ti_0.02_](OH)_2_K_0.78_Ca_0.01_Na_0.09_	2.05	− 1.02	+ 0.89
F1	Supplementary Fig. 1a	Points 1 to 18	[Si_3.07_Al_0.93_]O_10_[Al_1.78_ Fe_0.11_Mg_0.08_Ti_0.04_](OH)_2_K_0.77_Ca_0.01_Na_0.15_	2.01	− 0.97	+ 0.94
	Supplementary Fig. 1b	Points 1 to 12	[Si_3.07_Al_0.93_]O_10_[Al_1.81_ Fe_0.10_Mg_0.06_Ti_0.04_](OH)_2_K_0.69_Ca_0.01_Na_0.25_	2.01	− 0.95	+ 0.96
	Supplementary Fig. 1c	Points 1 to 10	[Si_3.06_Al_0.94_]O_10_[Al_1.75_ Fe_0.16_Mg_0.09_Ti_0.04_](OH)_2_K_0.72_Ca_0.01_Na_0.15_	2.04	− 0.99	+ 0.89
	Supplementary Fig. 1d	Points 1 to 9	[Si_3.05_Al_0.95_]O_10_[Al_1.82_ Fe_0.10_Mg_0.05_Ti_0.03_](OH)_2_K_0.67_Ca_0.00_Na_0.28_	2.00	− 0.97	+ 0.95
	Supplementary Fig. 1e	Points 1 to 14	[Si_3.09_Al_0.91_]O_10_[Al_1.73_ Fe_0.13_Mg_0.12_Ti_0.05_](OH)_2_K_0.82_Ca_0.00_Na_0.12_	2.03	− 0.98	+ 0.94
	Supplementary Fig. 1f	Points 1 to 9	[Si_3.07_Al_0.93_]O_10_[Al_1.74_ Fe_0.13_Mg_0.10_Ti_0.05_](OH)_2_K_0.79_Ca_0.00_Na_0.13_	2.02	− 0.98	+ 0.92
F3	Figure 3c	Point 15	[Si_3.21_Al_0.79_]O_10_[Al_1.62_ Fe_0.19_Mg_0.26_Ti_0.03_](OH)_2_K_0.70_Ca_0.01_Na_0.01_	2.10	− 1.02	+ 0.73
	Figure 3d	Point 1	[Si_3.19_Al_0.81_]O_10_[Al_1.56_ Fe_0.24_Mg_0.30_Ti_0.02_](OH)_2_K_0.71_Ca_0.01_Na_0.01_	2.12	− 1.09	+ 0.74
F1	Supplementary Fig. 2	Points 1 to 6	[Si_3.05_Al_0.95_]O_10_[Al_1.81_ Fe_0.11_Mg_0.06_Ti_0.03_](OH)_2_K_0.69_Ca_0.01_Na_0.25_	2.01	− 0.98	+ 0.96
	Theoretical muscovite^60^		[Si_3.00_Al_1.00_]O_10_[Al_2.00_](OH)_2_K_1.00_	2.00	− 1.00	+ 1.00
	Muscovite in a granite^41^		[Si_3.16_Al_0.84_]O_10_[Al_1.66_ Fe_0.07_Mg_0.28_Ti_0.05_](OH)_2_K_0.95_	2.06	− 1.07	+ 0.95

Chemical compositions of the half unit cell of a theoretical muscovite^59^ and of a muscovite in a granite^60^ are also given.

**Figure 2:**
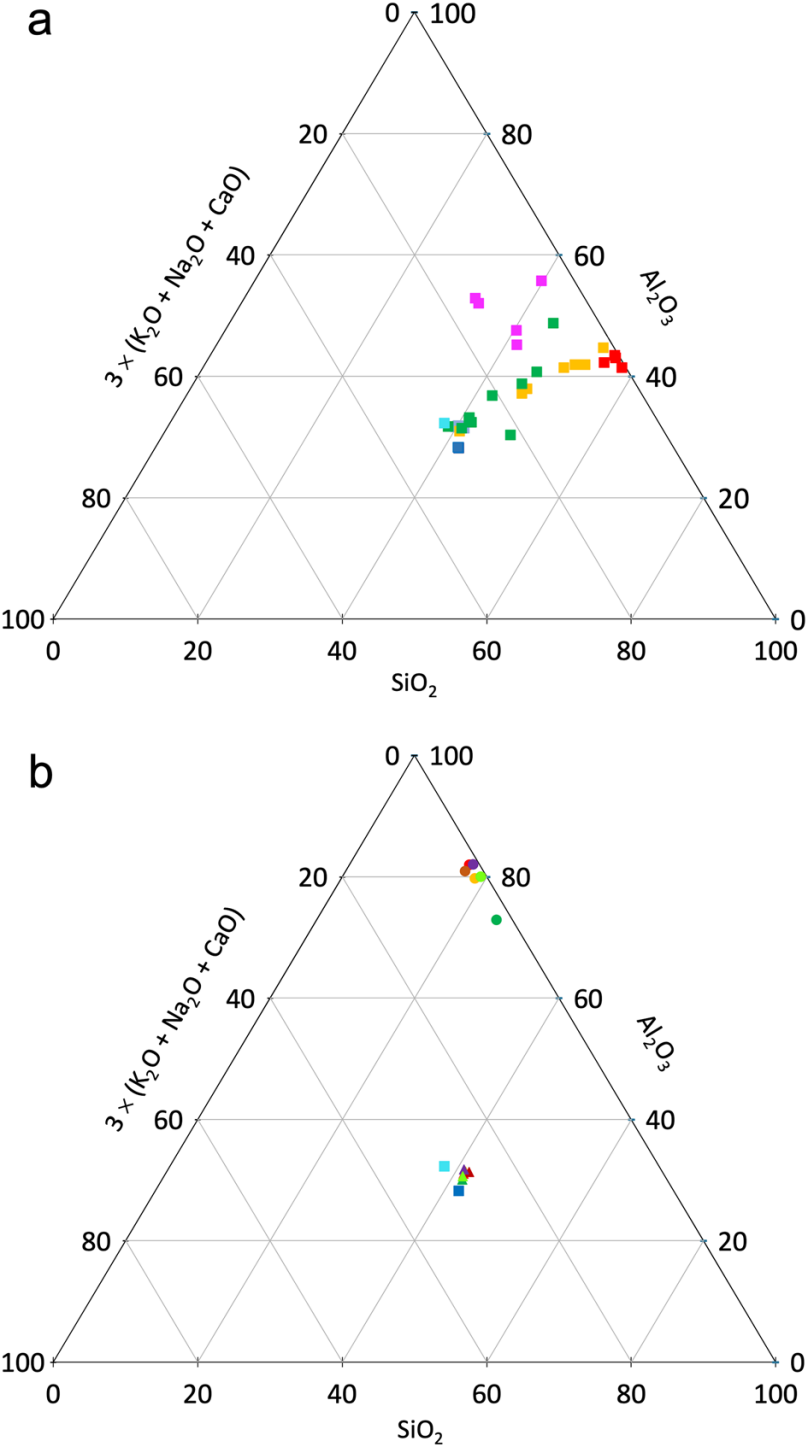
Respective content in AL_2_O_3_, SiO_2_ and K_2_O + Na_2_O + CaO in the elongated particles varying in size shown in Ferralsols F1 (grey squares, Fig. 1a), F2 (purple squares, Fig. 1c), F3 (yellow squares, Fig. 1e) and F4 (green squares, Fig. 1g). Theoretical composition of muscovite (light blue square)^59^, composition of muscovite particles (deep blue squares)^60^ and of weathered muscovite (red squares)^60^ are also plotted.

Comparison of the Al_2_O_3_, SiO_2_ and K_2_O + Na_2_O + CaO contents recorded for the elongated particles observed in F1, F2, F3 and F4 with their respective contents in a theoretical muscovite, an unweathered muscovite and a highly weathered muscovite showed that the chemical compositions recorded for the fine sand-size elongated particle shown in Fig. 1a and for the other elongated particles showing the highest K_2_O content (Fig. 1e,f, Supplementary Table 1) were close to those of the theoretical muscovite and unweathered muscovite (Fig. 2a). Figure 2a shows also that the zonation observed on the BESI of the particle analyzed in F3 and the map of its K_2_O content corresponded to a variation in the chemical composition between a weakly weathered muscovite and a deeply weathered muscovite. Lastly, the small-size elongated particles analyzed in Fig. 1c showed a smaller SiO_2_ content. This can be attributed to the fact that the particles analyzed were so small that the analysis partly included partly the surrounding groundmass material. this explains their location in the triangle showing the Al_2_O_3_, SiO_2_ and K_2_O + Na_2_O + CaO contents (Fig. 2a). Globally, the increase in the Al_2_O_3_ content when the K_2_O decreased has to be considered as the result of the intercalation of hydroxy-Al in the inter-layer space during the weathering process of the muscovite particles^18^ leading to the formation of hydroxy-Al interlayered vermiculites (HIV) as shown by several authors^29,31–37^.

The BESI of F1 showed the presence of many large fine sand-size elongated particles with phyllosilicate morphology similar to the particle shown in Fig. 1a (Supplementary Fig. 1, Table 1). They all exhibited an internal layer organization with some large inter-layer spaces resulting from swelling properties, depending on the chemical composition of the 2:1 layers. The collapse of most inter-layer spaces when drying to the benefit of the greater opening of some inter-layer spaces explains the large inter-layer spaces observed. The K_2_O content in these fine sand-size elongated particles ranged from 7.51 to 11.24% (Supplementary Fig. 1, Supplementary Table 2). Comparison of the Al_2_O_3_, SiO_2_ and K_2_O + Na_2_O + CaO mean contents recorded for these sand-size elongated particles and their respective contents in a theoretical muscovite and a natural unweathered muscovite showed that they were particles of very weakly weathered muscovite which can only originate from the underlying saprolite present from 300 cm depth (“Methods”) (Fig. 2b). The groundmass chemical composition (Fig. 2b, Supplementary Table 2) surrounding these sand-size particles is consistent with a mineralogical composition dominated by gibbsite as reported earlier for F1^38^, thus emphasizing the allochthonous character of the muscovite particles. We also computed the structural formula for these 2:1 clay minerals on the basis of the structure of a 2:1 phyllosilicate (Table 2). Results showed a very similar chemical composition of the half unit cell for the six particles analyzed, thus indicating that they originated from a saprolite containing a single type of muscovite with Na^+^ in the interlayer space representing from 17 to 30% of the interlayer electrical charge (Table 2).

### Allochthonous clay particles and associated fine material

**Figure 3:**
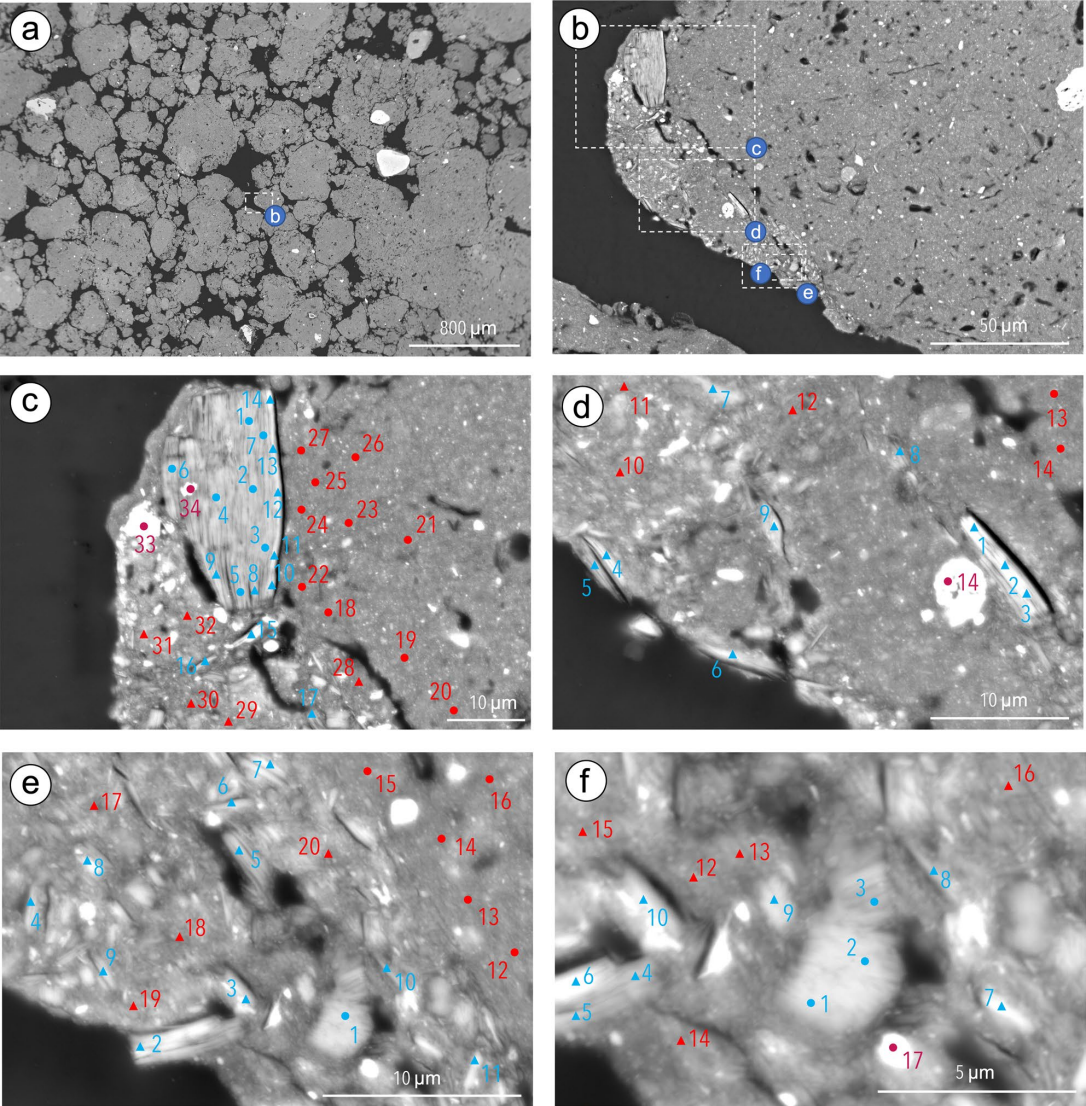
Location of an area with allochthonous material associated to the submillimetric granular aggregates (**a**) and of the four subareas where the analyses were performed (**b**) on the backscattered scanning images (BESI) of the cross sections of Ferralsols F3. Location of the analyses performed by using energy dispersive spectrometry (EDS) (**c**–**f**) in the particles with a small K_2_O content (blue circles), the particles with a high K_2_O content (blue triangles), their associated groundmass (red triangles), the groundmass of the surrounding submillimetric granular aggregates (red circles) and particles with a high Fe_2_O_3_ content (deep red circles). The numbers correspond to the analysis points in Supplementary Table 3.

Some areas with a groundmass that included a high proportion of elongated particles with K_2_O content > 0.5% and showed a chemical composition highly different from that of the groundmass forming the main part of the ferralic B horizon were observed in F1 and F3. An area associating a high number of elongated particles varying in size and K_2_O content to fine material with a chemical composition highly different from that of the groundmass material of the neighboring microaggregate was observed in F3 (Fig. 3, Supplementary Table 3). Some elongated particles showed a small mean K_2_O content (0.26%) (Supplementary Table 3) and may correspond to kaolinite particles or to HIV particles in which most K^+^ was removed and replaced by hydroxylated Al^3+^ as discussed earlier^18^. The other elongated particles showed a varying K_2_O content but always > 0.5% with a mean K_2_O content of 2.80% (Fig. 3, Supplementary Table 3). The structural formulas computed for the analyses with the highest K_2_O content showed fewer substitutions of Si^4+^ by Al^3+^ than recorded for the large particle shown in F3 (Fig. 1e, Table 2) and for all the other elongated particles analyzed (Table 2). The structural formulas computed are close to that of a natural muscovite (Table 2). And if we limit the number of octahedral sites occupied at two sites by distributing Mg^2+^ between the octahedral sites and the interlayer space, the negative electrical charges of the layer (− 0.92 and − 0.91 for the two points analyzed) are better equilibrated with the positive electrical charges (+ 0.93 and + 0.96, respectively) resulting from the cations located within the interlayer space. As for the fine material closely associated to these elongated particles, its Al_2_O_3_ and SiO_2_ mean contents were 38.31 and 43.16%, respectively while they were 48.83 and 27.39%, respectively for the groundmass material of the neighboring microaggregate (Supplementary Table 3).

In F1, small aggregates consisting of elongated particles with K_2_O content > 0.5% closely associated to fine material with a chemical composition highly different from that of the groundmass material of the surrounding submillimetric granular aggregates were present (Supplementary Fig. 2, Supplementary Table 4). While the Al_2_O_3_ and SiO_2_ mean contents were 54.73 and 11.45%, respectively for the groundmass of the submillimetric granular aggregates (Supplementary Table 4), they were 43.71 and 28.16%, respectively, for the fine material associated to the elongated particles with K_2_O content > 0.5%. We also observed also a smaller K_2_O content smaller in the groundmass material of the surrounding submillimetric granular aggregates (mean content of 0.11%) compared to that of the fine material (mean content of 1.34%) associated to the elongated particles with K_2_O content > 0.5% (Supplementary Fig. 2, Supplementary Table 4). We can hypothesize that in the fine material the K_2_O content resulted from the presence of very small elongated particles of 2:1 clay minerals rich in K_2_O. The structural formula computed was consistent with those already computed for the fine sand-size elongated particles which corresponded to weakly weathered muscovite (Table 2, Fig. 1a and Supplementary Fig. 1).

**Figure 4:**
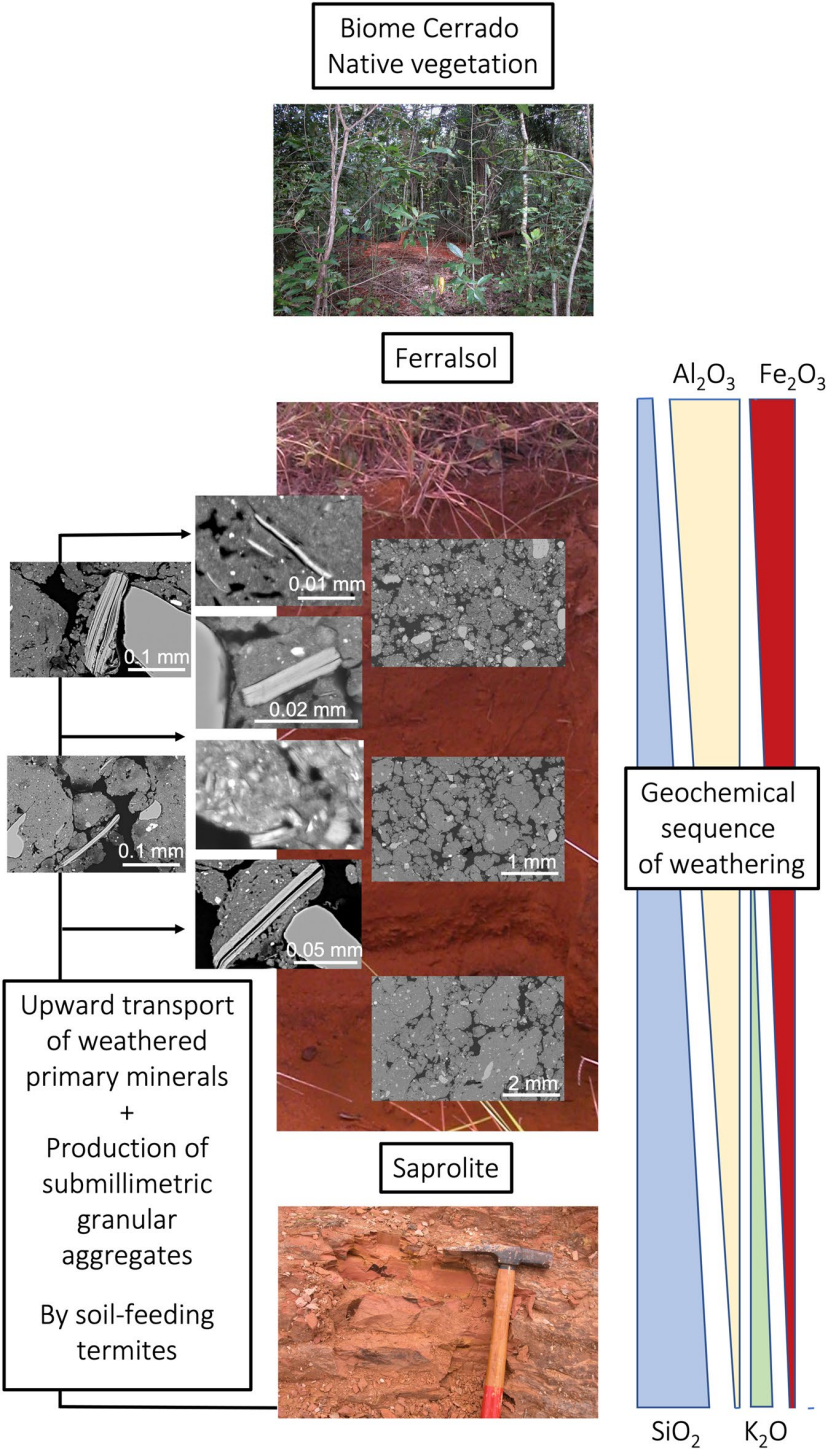
Synthetic diagram of the action of soil-feeding termites responsible for the presence of variably altered muscovite in the ferrallic B horizon of Ferralsols and of their submillimetric granular structure.

Thus, our results showed that fine material closely associated to the weathered muscovite particles had a smaller Al_2_O_3_ content and a higher SiO_2_ content than the fine material composing the groundmass of the surrounding submillimetric granular aggregates observed in F1 and F3. These smaller Al_2_O_3_ and higher SiO_2_ contents are additional arguments indicating that the muscovite particles and their associated fine material originate from the saprolite which is consistent with the geochemical sequence of weathering leading to the formation of Ferralsols^2–4^ (Fig. 4). Hence, the presence of weathered muscovite particles and their associated fine material have to be considered as markers of soil-feeding termite activity. We can also infer, as has been hypothesized by several authors^7,17,18,20,21,39^ (Fig. 4) that this ceaseless activity of soil-feeding termites consisting in fragmenting and reorganizing the soil is also the process responsible for the microaggregated structure of the Ferralsols.

## Implications

The implications of our results go far beyond the case of Ferralsols alone. If soil-feeding termites bring material from the underlying saprolite, usually located at several meters depth, up to the B horizons of Ferralsols, we can hypothesize that they bring similar material in all the horizons of Ferralsols and that they behave similarly for many other tropical soils^6,40–42^. In the latter, their potential contribution of material from the saprolite to the soil horizon is probably less visible than for Ferralsols due to a less sharp discontinuity in mineralogical composition between the soil and the underlying saprolite. This contribution of soil-feeding termite activity to the soil characteristics is probably more frequent than is generally thought, which is in agreement with studies performed by soil biologists on the mineralogical composition of termite mounds and of the immediately surrounding soils^19,40,43,44^.

The presence of weathered muscovite particles resulting from soil-feeding termite activity constitutes a source of available K^+^ to plant nutrition in Ferralsols and is as such partly responsible for their chemical fertility. The weathered muscovite particles have to be considered as markers of the soil-feeding termite activity. Another consequence of this activity is the submillimetric granular structure (Fig. 4) which is responsible for most of the physical properties of Ferralsols (e.g. water infiltration and retention, gas transfer properties, root penetration resistance). Consequently, when changes in land use occurs, more attention should be paid to possible changes affecting the soil-feeding termite communities^12,14,45^. Their contribution to both the physical and chemical properties of tropical soils is undoubtedly more important than is often acknowledged^44–46^. This is relevant to an issue currently under investigation by the scientific community, namely the consequences of the deforestation of savannas, particularly the sustainable management of these areas^13,14,47^.

## Methods

### The soils studied

The Ferralsols studied are located in the Brazilian Central Plateau which presents two main geomorphologic surfaces: the Late Tertiary South American Surface (usually 900 to 1200 m high) which corresponds to tablelands where gibbsitic-sesquioxidic Ferralsols are dominant and the Late Quaternary Velhas Surface, which occurs 5 to 25 m below the South American Surface and shows a moderate slope where kaolinitic-non-sesquioxidic Ferralsols are numerous^20,48–50^. The most representative climate of the Brazilian Central Plateau is Megathermic or Humid Tropical (Aw) with the savanna subtype^51^. It is characterized by maximum rains in summer and a dry winter (average temperature of the coldest month > 18 °C). The average annual rainfall ranges from 1500 to 2000 mm, with the highest amounts of rainfall occurring in January and the lowest from June to August (< 50 mm per month)^52^. Four ferralic B horizons belonging to four Ferralsols were selected among Ferralsols studied earlier^10,20,34,38,53–55^. The four Ferralsols selected were located under native Cerrado, three on the Velhas Surface (F1, F3 and F4) and one the South American Surface (F2). F1 was collected in a Ferralsol (S 16°38′50.84″ W 49°28′59.84″) located at an elevation of 739 m and developed on a saprolite of a Precambrian mafic granulite^56,57^ visible at a depth of 350 to 400 cm. The soil was originally recognized as being developed on the South American Surface^53^ but further field analysis showed that it was developed on the Velhas Surface. This Ferralsol was studied earlier (soil L1)^10,57^ and F1 corresponds to the horizon Bw_2_ (100–160 cm) of Ferralsol classified as a gibbsitic-sesquioxidic Rhodic Ferralsol^1^ (1.7 × [SiO_2_/Al_2_O_3_] = 0.4, Kaolinite/[Kaolinite + Gibbsite] = 0.3 and [Hematite/Hematite + Goethite] = 0.7). F2 was collected in a Ferralsol (S 15°36′3.20″ W 47°44′1.48″) located at an elevation of 1180 m and developed on a saprolite of a Precambrian quartzite^56,57^ visible at a depth of 200 to 250 cm. This Ferralsol was studied earlier (soil L4)^10,57^ and F2 corresponds to the horizon Bw_1_ (60–110 cm) of this soil classified as a gibbsitic-sesquioxidic Plinthic Ferralsol^1^ (1.7 × [SiO_2_/Al_2_O_3_] = 0.3, Kaolinite/[Kaolinite + Gibbsite] = 0.2 and [Hematite/Hematite + Goethite] = 0). F3 was collected in a Ferralsol (S 15°31′4.50″ W 47°41′9.03″) located at an elevation of 880 m and developed on a the saprolite of a Precambrian metapelite where a lateritic crust is present^56,57^ visible at a depth of 500 to 600 cm. This Ferralsol was studied earlier (soil L6)^10,53^ and F3 corresponds to the horizon Bw_2_ (140–200 cm) of a soil classified as kaolinitic-sesquioxidic Rhodic Ferralsol^1^ (1.7 × [SiO_2_/Al_2_O_3_] = 0.8, Kaolinite/[Kaolinite + Gibbsite] = 0.5 and [Hematite/Hematite + Goethite] = 0.5). Finally, F4 was collected in a Ferralsol located in the Mato Grosso state at an elevation of 430 m (S 21°21′59.96″ W 52°10′59.88″) and developed on a saprolite of Cretaceous sandstones (Baurú group) visible at a depth of 300 to 350 cm^54,55^. This Bw horizon corresponds to the 60–110 cm horizon of a soil classified as a Rhodic Ferralsol^1^. The four ferralic B horizons selected show a weak macrostructure and a strong submillimetric granular structure^10,54,55^. The clay content of F1, F2, F3 and F4 was 520, 300, 780 and 782 g kg^−1^, respectively. Their cation exchange capacity was 2.0, 1.7, 10.0 and 2.5 cmol_c_ kg^−1^, respectively with most exchangeable cations corresponding to H^+^ and Al^3+^^53,54^.

### Scanning electron microscopy and energy dispersive spectroscopy

Undisturbed samples were collected with 100-cm^3^ volume cylinders, dried and then included in a polyester resin^58^. After polymerization and hardening, circular cross sections 2.5 cm in diameter were prepared and carbon coated for examination in scanning electron microscopy (SEM) using backscattered electron scanning images (BESI). Observations were made from low magnifications ranging (35× to 200×) to high magnification ranging (1000× to 8000×) to identify particles of phyllosilicates in the groundmass^18^. The scanning electron microscope (SEM) used was a Merlin Compact Zeiss microscope (resolution of 0.8 nm at 15 kV and 1.6 nm at 1 kV; tension ranging from 20 V to 30 kV; probe current ranging from 12 pA to 100 nA). It was equipped with a Gemini I column including a backscattered electron detector (BSD) with five quadrants for acquisition of the backscattered electron scanning images (BESI). Observations were performed at 15 kV accelerating voltage and at a working distance of 10 mm. Chemical analyses were performed using energy dispersive X-ray spectroscopy (EDS) with a Quantax XFlash6 Bruker detector enabling a resolution of 129 eV. Analyses were performed also at 15 kV accelerating voltage. The SEM was operated with a resolution of 0.8 nm and a probe current of 1.6 nA. A count time of 100 s was used for punctual analyses. Total chemical composition was expressed on the basis that the sum of oxide mass equals 100 for determinations of SiO2, Al2O3, Fe2O3, MgO, CaO, K2O, and Na2O and TiO_2_. The chemical compositions were also plotted in ternary plots to show the Al_2_O_3_, SiO_2_ and K_2_O + Na_2_O + CaO contents. Images of the concentration of K throughout the images were recorded with an acquisition time of 15 min.

## Supplementary Information

Supplementary Information 1.

Supplementary Information 2.

## Data Availability

The datasets used during the current study are available from the corresponding author on reasonable request.
