# Supplementary material for: Allochthonous material originating from saprolite as a marker of termite activity in Ferralsols
Source: Sci Rep. 2022 Oct 13;12:17193. doi: 10.1038/s41598-022-21613-6 (PMC9562979; doi:10.1038/s41598-022-21613-6)
Supplement: Supplementary file 1 — Supplementary Information 1. [file 41598_2022_21613_MOESM1_ESM.pdf]

Supplementary material

Allochthonous material originating from saprolite as a marker of termite activity in Ferralsols

Ary Bruand *[,a], Adriana Reatto [b] and Éder de Souza Martins [c]

[a] Institut des Sciences de la Terre d'Orléans (ISTO) UMR7327, Université d'Orléans – CNRS – BRGM, Observatoire des Sciences de l'Univers en région Centre (OSUC), Campus Géosciences, Université d'Orléans, 1A rue de la Férollerie, 45071 Orléans, Cedex 2, France.

[b] Empresa Brasileira de Pesquisa Agropecuária (Embrapa), Secretaria de Pesquisa e Desenvolvimento, Parque Estação Biológica-PqEB s/n^0, Brasília-DF, Brazil.

[c] Empresa Brasileira de Pesquisa Agropecuária (Embrapa Cerrados), Brasília-DF, Brasil.

* Corresponding author: Ary Bruand (Ary.Bruand@univ-orleans.fr)

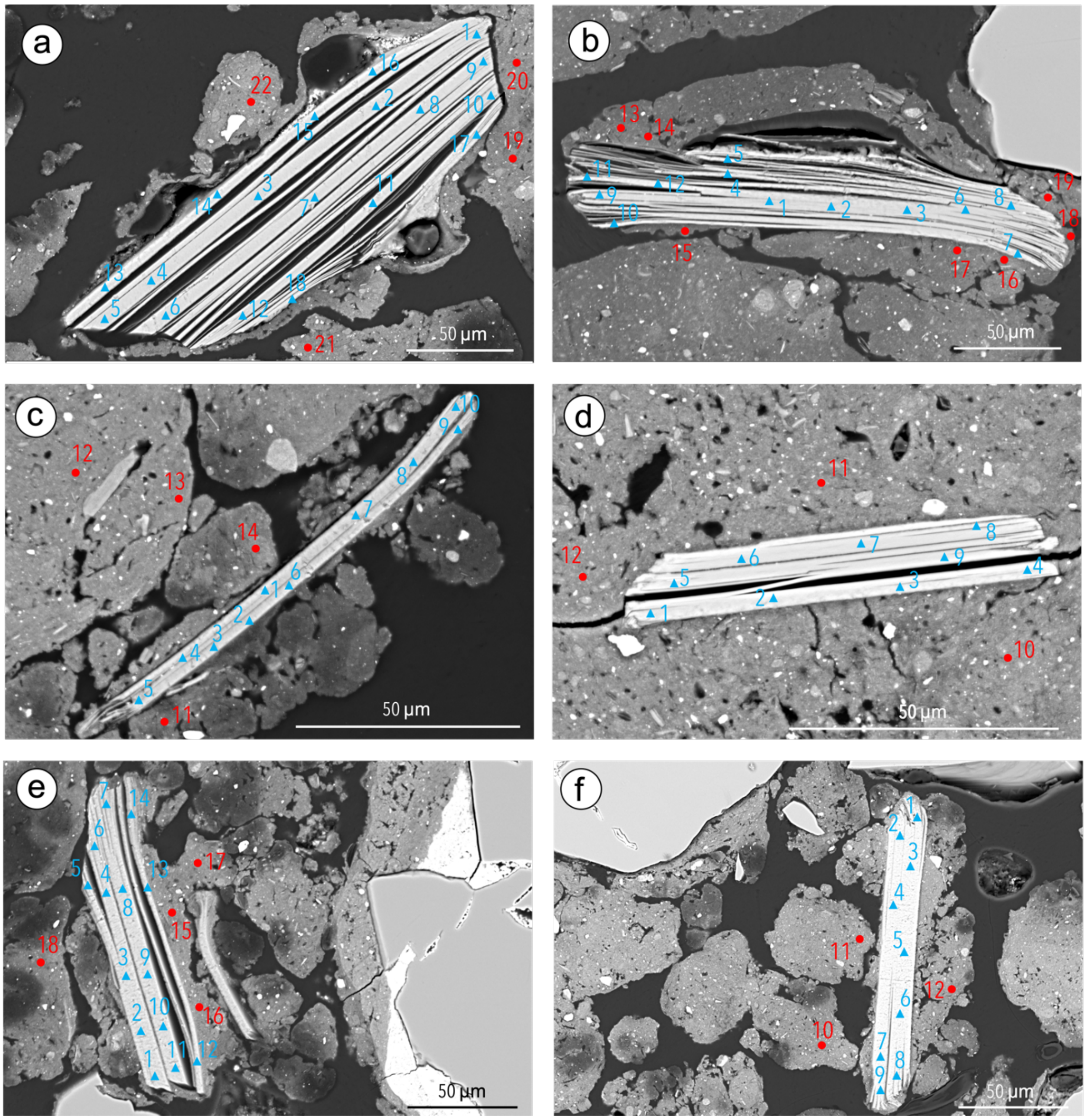

Supplementary Fig. 1 | Elongated large particles observed on the backscattered scanning images (BESI) of the cross sections of F1, location of the analyses performed by using energy dispersive spectrometry (EDS) in the particles (blue triangle) and their surrounding groundmass (red circles). The numbers correspond to the analysis points in Supplementary Table 2.

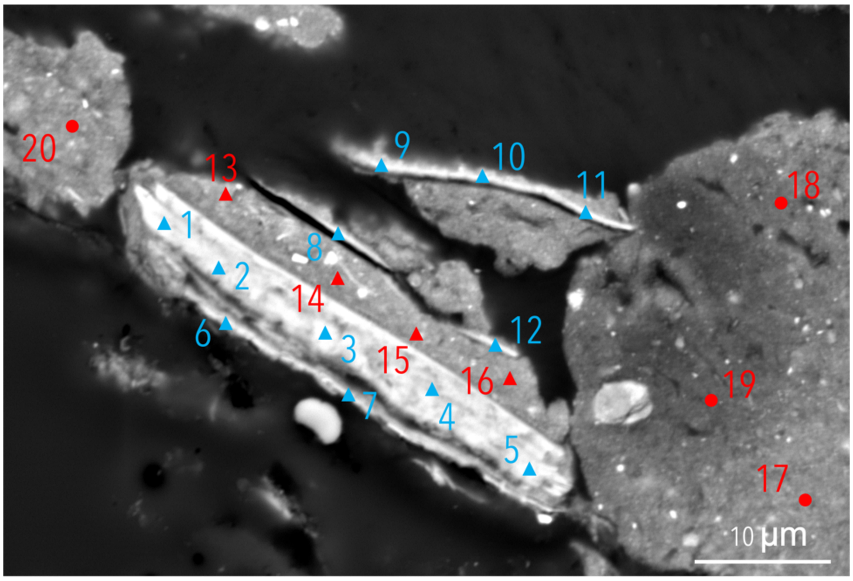

Supplementary Fig. 2 | Area with allochthonous material associating elongated particles and groundmass with chemical composition different from that of the surrounding submillimetric granular aggregates on the backscattered scanning images (BESI) of the cross sections of F1. Location of the analyses performed by using energy dispersive spectrometry (EDS) in particles with a high K_2O content (blue triangles), their associated groundmass (red triangles), and the groundmass of the surrounding submillimetric granular aggregates (red circles). The numbers correspond to the analysis points in Supplementary Table 4.

Supplementary Table 1 | Chemical analyses of the particles shown in the backscattered scanning images (BESI) of the four Ferralsols studied (Fig. 1) using energy dispersive spectroscopy (EDS).

N° of analysis	% of oxides							
	Al_2O_3	SiO_2	Fe_2O_3	MgO	CaO	K_2O	Na_2O	TiO_2
Particle in Fig 1a & 1b – F1								
1	37.57	48.02	2.14	0.80	0.00	8.74	2.15	0.58
2	37.94	47.67	2.01	0.59	0.05	9.05	2.04	0.65
3	37.84	47.63	2.00	0.59	0.00	9.17	2.18	0.59
4	36.84	48.19	2.33	0.99	0.06	8.74	1.90	0.96
Particles in Fig 1c & 1d – F2								
1	43.39	39.87	9.17	1.14	0.03	4.17	0.04	2.19
2	43.56	36.95	12.85	0.98	0.06	3.58	0.07	1.95
3	46.72	29.49	16.96	0.59	0.00	4.46	0.05	1.73
4	49.00	29.62	14.57	0.68	0.00	4.57	0.12	1.44
5	49.99	35.59	12.32	0.26	0.03	1.33	0.01	0.48
Particle in Fig 1e & 1f – F3								
1	40.60	49.83	2.76	0.55	0.00	5.36	0.16	0.75
2	35.66	46.91	5.11	0.68	0.10	10.26	0.50	0.79
3	42.66	53.63	1.55	0.07	0.00	1.84	0.00	0.25
4	39.57	49.21	4.07	0.67	0.06	5.66	0.14	0.62
5	42.86	51.73	1.91	0.27	0.05	2.83	0.09	0.26
6	42.95	52.47	1.84	0.26	0.05	2.27	0.02	0.13
7	44.29	53.28	1.83	0.05	0.01	0.50	0.00	0.05
Particles in Fig 1g & 1h – F4								
1	37.58	48.25	3.27	0.55	0.04	9.30	0.64	0.36
2	38.54	47.59	2.61	0.50	0.13	9.06	0.80	0.76
3	38.21	46.74	2.47	0.38	0.15	10.87	0.81	0.36
4	38.15	47.71	3.33	0.46	0.03	9.32	0.74	0.25
5	36.23	47.06	4.92	0.82	0.16	9.83	0.69	0.29
6	26.63	42.20	21.21	2.99	0.05	6.25	0.01	0.67
7	39.76	45.78	5.92	0.57	0.07	6.85	0.58	0.47
8	38.41	44.99	9.36	0.82	0.04	5.08	0.07	1.22
9	38.33	43.80	12.06	0.88	0.00	3.93	0.06	0.93
9	36.85	33.85	13.74	0.61	0.06	1.37	0.19	13.32
10	37.69	43.39	9.65	0.75	0.08	6.18	0.41	1.85

Supplementary Table 2 | Chemical analyses of the elongated large particles shown in the backscattered scanning images (BESI) of F1 and of their surrounding groundmass (Supplementary Fig. 1) using energy dispersive spectroscopy (EDS).

Analysis number	% of oxides							
	Al_2O_3	SiO_2	Fe_2O_3	MgO	CaO	K_2O	Na_2O	TiO_2
	Particle in Supplementary Fig. 1a							
1	36.98	48.42	2.48	0.79	0.10	9.08	1.22	0.93
2	36.29	48.29	2.13	0.85	0.11	10.06	1.27	1.01
3	35.53	47.76	2.39	0.85	0.20	11.24	1.29	0.74
4	36.04	49.16	2.17	0.93	0.08	9.53	1.31	0.78
5	36.60	48.82	2.10	0.96	0.07	9.35	1.28	0.80
6	36.04	48.85	2.41	0.88	0.11	9.65	1.26	0.80
7	36.66	48.33	2.16	0.93	0.12	9.70	1.36	0.75
8	36.30	48.27	2.54	0.87	0.10	9.55	1.31	1.06
9	37.01	48.60	2.77	0.74	0.04	8.84	1.14	0.87
10	36.35	48.83	2.49	0.69	0.05	9.35	1.13	1.10
11	35.97	49.01	2.35	0.96	0.09	9.60	1.22	0.80
12	36.82	48.92	2.43	0.84	0.01	8.81	1.35	0.83
13	35.70	49.00	2.15	0.83	0.07	9.96	1.32	0.97
14	36.98	47.89	2.20	0.92	0.20	9.70	1.41	0.69
15	36.11	48.66	2.77	0.78	0.02	9.26	1.32	1.07
16	35.72	48.00	2.66	0.93	0.03	10.03	1.38	1.24
17	37.53	48.78	2.72	0.81	0.09	8.24	1.18	0.64
18	37.34	48.66	2.47	1.08	0.11	8.85	0.96	0.53
Mean	36.44	48.57	2.41	0.87	0.09	9.49	1.26	0.87
Standard deviation	0.58	0.41	0.22	0.09	0.05	0.65	0.11	0.18
	Surrounding groundmass in Supplementary Fig. 1a							
19	56.41	11.99	28.53	0.14	0.00	0.63	0.12	2.18
20	53.59	11.21	33.14	0.01	0.04	0.00	0.00	2.01
21	55.00	11.07	29.78	0.01	0.07	0.12	0.13	3.81
22	58.95	10.97	27.65	0.03	0.03	0.11	0.06	2.20
Mean	55.99	11.31	29.78	0.05	0.04	0.21	0.08	2.55
Standard deviation	2.29	0.47	2.41	0.06	0.03	0.28	0.06	0.84
	Particle in Supplementary Fig. 1b							
1	36.66	48.97	2.07	0.60	0.07	8.80	2.06	0.77
2	36.70	49.29	1.97	0.59	0.01	8.72	1.85	0.88
3	35.46	49.26	1.96	0.62	0.09	9.91	1.91	0.79
4	37.17	49.01	2.08	0.52	0.08	8.38	2.15	0.62
5	36.84	49.28	2.01	0.56	0.00	8.40	2.12	0.79
6	38.11	48.10	1.89	0.76	0.02	8.39	2.06	0.65
7	37.97	48.30	2.21	0.63	0.16	8.54	1.66	0.52
8	37.36	49.12	2.11	0.66	0.07	8.35	1.75	0.58
9	37.57	49.19	2.00	0.50	0.00	7.51	2.39	0.85
10	36.34	47.98	3.31	0.58	0.11	8.52	2.12	1.05
11	36.49	48.85	2.35	0.57	0.10	8.38	2.38	0.88
12	36.78	48.60	2.08	0.54	0.13	8.72	2.45	0.71
Mean	36.95	48.83	2.17	0.59	0.07	8.55	2.08	0.76
Standard deviation	0.74	0.47	0.38	0.07	0.05	0.54	0.25	0.15

			Surrounding groundmass in Supplementary Fig. 1b					
13	52.73	12.20	31.55	0.00	0.06	0.53	0.09	2.83
14	49.44	11.64	27.86	0.15	0.10	0.19	0.04	10.57
15	53.99	11.60	32.02	0.08	0.00	0.12	0.11	2.08
16	49.89	11.16	33.96	0.10	0.00	0.25	0.07	4.57
17	54.60	11.70	30.47	0.07	0.06	0.29	0.04	2.77
18	52.75	13.44	31.34	0.08	0.07	0.18	0.06	2.08
19	48.62	11.99	27.82	0.09	0.09	0.30	0.10	10.99
Mean	51.99	12.38	29.87	0.08	0.07	0.26	0.07	5.28
Standard deviation	3.06	0.93	1.83	0.01	0.01	0.07	0.03	4.96
			Particle in Supplementary Fig. 1c					
	36.57	48.18	2.83	0.90	0.12	9.17	1.47	0.77
	36.06	48.58	2.85	0.80	0.18	9.48	1.27	0.78
	35.44	48.91	3.38	0.87	0.08	9.07	1.30	0.94
	36.45	48.97	2.31	0.87	0.11	9.20	1.43	0.67
	36.52	49.47	2.86	1.30	0.01	8.50	0.78	0.55
	35.72	49.40	2.76	1.21	0.12	9.22	0.88	0.69
	36.47	48.10	2.65	0.91	0.02	9.66	1.42	0.77
	35.84	45.28	7.57	0.86	0.15	7.87	1.35	1.07
	36.57	49.57	2.51	0.74	0.11	8.56	1.35	0.60
Mean	36.18	48.50	3.30	0.94	0.10	8.97	1.25	0.76
Standard deviation	0.43	1.32	1.63	0.19	0.06	0.56	0.25	0.16
			Surrounding groundmass in Supplementary Fig. 1c					
	61.09	10.32	25.78	0.03	0.19	0.45	0.00	2.14
	52.15	12.34	29.64	0.04	0.11	0.77	0.10	4.84
	54.41	10.81	30.34	0.06	0.19	0.16	0.04	3.99
	51.78	11.36	34.38	0.01	0.07	0.12	0.08	2.20
Mean	54.86	11.21	30.04	0.03	0.14	0.38	0.06	3.29
Standard deviation	4.31	0.87	3.52	0.02	0.06	0.30	0.05	1.34
			Particle in Supplementary Fig. 1d					
	37.74	48.83	2.15	0.49	0.06	7.79	2.25	0.69
	38.28	48.01	1.90	0.48	0.07	7.94	2.63	0.68
	37.77	48.41	1.99	0.44	0.01	8.12	2.37	0.90
	36.23	48.98	2.84	0.70	0.05	9.25	1.53	0.41
	37.18	48.57	2.39	0.50	0.02	8.32	2.38	0.64
	37.46	48.56	2.02	0.48	0.08	8.24	2.60	0.55
	38.07	47.48	2.02	0.54	0.04	8.30	2.85	0.69
	36.88	48.63	2.14	0.61	0.07	8.97	2.03	0.67
	37.65	48.57	2.14	0.59	0.07	8.60	1.99	0.38
Mean	37.47	48.45	2.18	0.54	0.05	8.39	2.29	0.63
Standard deviation	0.63	0.45	0.29	0.08	0.03	0.47	0.40	0.16
			Surrounding groundmass in Supplementary Fig. 1d					
	59.07	10.30	28.74	0.07	0.02	0.02	0.00	1.77
	54.81	12.21	29.17	0.14	0.03	0.14	0.02	3.47
	51.59	11.94	32.23	0.05	0.01	0.26	0.06	3.87
Mean	55.16	11.48	30.05	0.09	0.02	0.14	0.03	3.04
Standard deviation	3.76	1.03	1.91	0.05	0.01	0.12	0.03	1.12

	Particle in Supplementary Fig. 1e							
	34.67	49.28	2.56	1.29	0.00	10.01	0.90	1.30
	35.15	48.22	2.58	1.35	0.04	10.48	1.00	1.19
	35.13	48.31	2.64	1.28	0.07	10.73	0.99	0.86
	35.02	48.83	2.61	1.21	0.02	10.28	1.09	0.95
	35.84	47.53	3.55	1.19	0.00	9.86	1.03	1.01
	35.23	49.14	2.65	1.17	0.01	10.02	0.86	0.90
	36.48	48.73	2.91	0.97	0.03	9.15	0.93	0.79
	35.47	48.35	2.67	1.20	0.02	10.19	1.04	1.05
	34.81	49.01	2.61	1.19	0.08	10.29	0.96	1.05
	35.65	48.38	2.22	1.32	0.13	10.32	1.00	0.97
	34.23	49.29	2.58	1.27	0.01	10.50	0.88	1.24
	33.89	48.85	2.83	1.27	0.11	10.92	0.81	1.31
	35.17	48.36	2.82	1.23	0.00	10.28	1.07	1.08
	35.90	50.39	2.85	1.04	0.08	8.19	0.84	0.70
Mean	35.19	48.76	2.72	1.21	0.04	10.09	0.96	1.03
Standard deviation	0.68	0.67	0.29	0.10	0.04	0.69	0.09	0.19
	Surrounding groundmass in Supplementary Fig. 1e							
	48.05	23.30	25.39	0.36	0.11	0.85	0.04	1.91
	48.62	28.32	21.03	0.16	0.03	0.68	0.02	1.13
	61.79	10.45	24.81	0.05	0.06	0.14	0.00	2.71
	52.91	10.15	34.85	0.05	0.01	0.09	0.08	1.85
Mean	52.84	18.06	26.52	0.16	0.05	0.44	0.04	1.90
Standard deviation	6.35	9.19	5.88	0.14	0.04	0.38	0.03	0.65
	Particle in Supplementary Fig. 1f							
	35.85	48.29	2.88	1.03	0.06	9.71	1.09	1.09
	35.42	48.63	2.44	1.10	0.01	10.29	1.06	1.05
	35.73	48.46	2.57	0.98	0.12	10.06	1.09	0.99
	35.65	48.91	2.37	0.89	0.01	9.91	1.03	1.24
	36.22	48.42	2.40	0.97	0.02	9.72	1.13	1.14
	35.50	48.89	2.60	0.93	0.00	9.88	1.10	1.10
	35.59	49.03	2.65	0.99	0.04	9.45	1.12	1.15
	35.25	48.31	2.98	1.12	0.02	10.23	1.04	1.04
	37.26	47.58	3.08	1.06	0.04	9.02	1.08	0.88
Mean	35.83	48.50	2.66	1.01	0.03	9.81	1.08	1.08
Standard deviation	0.60	0.44	0.26	0.08	0.04	0.40	0.03	0.10
	Surrounding groundmass in Supplementary Fig. 1f							
	39.34	8.05	50.72	0.01	0.09	0.11	0.00	1.69
	49.17	12.49	35.48	0.08	0.07	0.11	0.03	2.58
	55.07	13.86	28.78	0.56	0.08	0.00	0.03	1.63
Mean	47.86	11.47	38.32	0.21	0.08	0.07	0.02	1.97
Standard deviation	7.95	3.03	11.24	0.30	0.01	0.06	0.02	0.53

Supplementary Table 3 | Chemical analyses using energy dispersive spectroscopy (EDS) of the elongated particles and their associated fine material, and of the groundmass of the submillimetric granular aggregate to which the area with many elongated particles was attached as shown in the BESI of F3 (Fig. 3).

Location of the analysis	N° of analysis	% of oxides							
		Al_2O_3	SiO_2	Fe_2O_3	MgO	CaO	K_2O	Na_2O	TiO_2
		Elongated particles with K_2O < 0.5 % - Blue circles							
Fig. 3a	1	44.19	54.17	1.18	0.03	0.00	0.35	0.00	0.07
	2	42.48	55.40	1.68	0.06	0.09	0.19	0.00	0.10
	3	44.48	53.16	1.57	0.15	0.01	0.56	0.05	0.01
	4	42.59	55.61	1.58	0.01	0.15	0.05	0.00	0.00
	5	42.58	54.67	2.20	0.06	0.00	0.35	0.00	0.13
	6	42.09	54.69	2.76	0.07	0.13	0.20	0.00	0.07
	7	42.73	55.16	1.44	0.09	0.00	0.45	0.08	0.05
Fig. 3e	1	41.29	56.27	2.09	0.07	0.03	0.08	0.00	0.19
Fig. 3f	1	43.04	52.87	3.57	0.15	0.00	0.15	0.04	0.18
	2	43.69	53.76	1.97	0.20	0.04	0.22	0.09	0.04
	3	41.79	51.11	5.60	0.27	0.00	0.49	0.09	0.65
	Mean	42.81	54.26	2.33	0.11	0.04	0.28	0.03	0.14
	Standard deviation	0.98	1.47	1.28	0.08	0.06	0.17	0.04	0.18
		Elongated particles with K_2O > 0.5 % - Blue triangles							
Fig. 3c	8	43.68	53.03	2.48	0.10	0.00	0.68	0.03	0.01
	9	41.90	54.52	2.14	0.14	0.00	0.94	0.05	0.31
	10	43.06	54.92	1.50	0.01	0.00	0.51	0.00	0.00
	11	42.68	52.45	1.84	0.37	0.00	2.30	0.04	0.32
	12	43.20	53.37	1.76	0.18	0.00	1.41	0.03	0.05
	13	43.74	53.74	1.42	0.12	0.04	0.88	0.06	0.00
	14	43.55	53.73	1.50	0.23	0.02	0.88	0.01	0.08
	15	32.61	51.05	4.02	2.70	0.17	8.67	0.08	0.69
	16	34.88	48.48	10.13	1.23	0.04	4.46	0.01	0.78
	17	37.37	53.05	4.70	1.49	0.03	3.08	0.06	0.22
Fig. 3d	1	31.83	50.52	5.03	3.20	0.12	8.81	0.04	0.44
	2	35.97	48.59	7.20	1.76	0.00	5.48	0.10	0.90
	3	36.75	49.57	7.13	1.41	0.08	4.06	0.06	0.93
	4	40.09	51.13	5.29	0.35	0.00	2.58	0.25	0.31
	5	40.63	50.15	6.87	0.33	0.00	1.36	0.15	0.51
	6	42.00	49.89	5.54	0.51	0.09	1.52	0.02	0.43
	9	38.70	44.18	12.22	0.46	0.03	1.51	0.06	2.84
	8	40.64	44.88	11.34	0.57	0.01	1.76	0.00	0.80
	7	33.42	47.30	11.54	2.18	0.17	4.55	0.04	0.81
Fig. 3e	2	32.47	45.33	15.00	1.63	0.05	4.61	0.02	0.87
	5	41.90	50.34	6.52	0.25	0.08	0.75	0.03	0.14
	6	37.67	45.78	14.20	0.43	0.01	1.30	0.04	0.57
	7	37.36	49.23	8.59	0.87	0.00	3.51	0.05	0.38
	4	39.01	46.28	7.99	1.75	0.00	4.04	0.06	0.87
	3	40.12	48.45	9.99	0.28	0.00	0.68	0.03	0.46
	8	32.75	49.96	7.34	2.91	0.00	6.58	0.04	0.43
	9	42.21	50.03	6.30	0.22	0.02	0.61	0.04	0.56

	10	37.62	47.14	11.99	0.58	0.00	2.10	0.09	0.48
	11	34.37	51.65	6.91	1.80	0.02	4.77	0.03	0.44
Fig. 3f	4	42.15	43.16	10.32	0.60	0.07	1.54	0.18	1.98
	5	40.89	40.96	14.60	0.55	0.08	1.94	0.10	0.86
	6	38.24	45.04	13.44	0.64	0.00	2.14	0.17	0.33
	7	35.06	46.45	11.34	0.82	0.12	5.38	0.11	0.73
	8	38.69	48.13	9.75	0.66	0.01	1.94	0.01	0.80
	9	41.39	50.97	5.92	0.33	0.00	0.58	0.09	0.73
	10	41.11	49.35	8.20	0.24	0.00	0.69	0.07	0.35
	Mean	38.74	49.14	7.70	0.91	0.04	2.80	0.06	0.61
	Standard deviation	3.60	3.38	4.00	0.85	0.05	2.25	0.05	0.54
		Fine material associated to the elongated particles – Red triangles							
Fig. 3c	30	38.33	39.70	19.10	0.46	0.05	0.90	0.07	1.38
	28	45.64	33.01	20.30	0.12	0.05	0.08	0.08	0.71
	29	38.42	43.12	14.98	0.55	0.04	1.26	0.15	1.49
	32	38.47	48.89	11.39	0.11	0.16	0.19	0.05	0.74
	31	38.02	47.46	12.38	0.45	0.01	1.12	0.05	0.50
Fig. 3d	12	40.32	49.33	8.69	0.23	0.00	0.67	0.08	0.67
	10	38.66	41.32	15.88	0.44	0.06	0.98	0.12	2.54
	11	37.85	44.08	13.94	0.32	0.07	0.80	0.03	2.91
Fig. 3e	17	34.99	44.13	15.67	0.75	0.06	2.15	0.06	2.18
	18	37.99	45.20	14.12	0.27	0.06	0.95	0.13	1.26
	20	35.93	40.51	17.59	0.40	0.13	1.01	0.15	4.29
	21	38.58	43.13	16.05	0.37	0.26	0.74	0.00	0.87
Fig. 3f	12	36.10	43.73	18.38	0.44	0.00	1.32	0.03	1.56
	13	34.65	39.99	21.83	0.44	0.15	1.27	0.04	1.52
	14	40.75	43.73	13.94	0.28	0.09	0.48	0.08	1.83
	15	38.21	46.10	13.17	0.42	0.00	1.13	0.05	0.55
	16	41.36	44.27	13.08	0.35	0.03	0.87	0.05	0.92
	Mean	38.31	43.16	15.62	0.38	0.08	0.93	0.08	1.63
	Standard deviation	2.67	4.07	3.47	0.16	0.07	0.50	0.05	1.02
		Groundmass material of submillimetric granular aggregate – Red circles							
Fig. 3a	18	45.65	26.32	25.40	0.14	0.09	0.06	0.12	1.01
	19	47.94	23.96	27.07	0.08	0.08	0.03	0.12	2.09
	20	46.69	32.64	17.78	0.05	0.10	0.06	0.15	0.76
	21	48.76	25.27	24.06	0.00	0.23	0.03	0.10	2.53
	22	47.12	30.10	21.17	0.04	0.01	0.18	0.03	0.81
	23	49.46	28.08	20.58	0.05	0.00	0.04	0.10	1.66
	24	53.42	28.08	17.44	0.15	0.02	0.07	0.13	1.44
	25	52.47	26.61	19.71	0.07	0.13	0.01	0.08	2.18
	26	50.73	28.56	18.79	0.19	0.03	0.03	0.07	1.22
	27	47.66	28.43	21.96	0.06	0.14	0.10	0.04	1.48
Fig. 3d	13	48.29	25.82	25.65	0.10	0.00	0.02	0.12	1.01
	14	51.04	28.11	19.75	0.02	0.02	0.00	0.03	1.77
Fig. 3e	12	45.58	25.91	25.24	0.09	0.00	0.01	0.07	1.01
	13	47.73	29.55	20.46	0.05	0.00	0.11	0.11	3.27
	14	49.32	31.04	17.51	0.12	0.00	0.17	0.10	1.90
	15	49.87	23.19	25.03	0.05	0.11	0.06	0.00	1.71
	Mean	48.83	27.39	22.00	0.08	0.06	0.06	0.08	1.62
	Standard deviation	2.19	2.61	3.34	0.05	0.07	0.06	0.04	0.66

		Subrounded shiny particles – Deep red circles							
Fig. 3c	33	3.86	4.04	90.94	0.04	0.05	0.13	0.24	0.70
	34	4.17	4.57	89.87	0.11	0.09	0.19	0.13	0.87
Fig. 3d	14	9.79	3.22	85.31	0.41	0.02	0.04	0.81	0.41
Fig. 3f	17	15.74	17.52	60.16	0.13	0.13	0.39	0.05	5.88
	Mean	8.39	7.34	81.57	0.17	0.07	0.18	0.31	1.97
	Standard deviation	5.61	6.81	14.48	0.16	0.05	0.15	0.35	2.62

Supplementary Table 4 | Chemical analyses of the particles and their associated fine material and of the groundmass of the surrounding submillimetric granular aggregates shown in the BESI of F1 (Supplementary Fig. 2) using energy dispersive spectroscopy (EDS).

N° of analysis	% of oxides							
	Al_2O_3	SiO_2	Fe_2O_3	MgO	CaO	K_2O	Na_2O	TiO_2
	Large elongated particle – Blue triangles							
1	38.53	48.36	2.00	0.56	0.06	7.62	2.20	0.67
2	36.74	48.26	2.70	0.56	0.07	8.95	2.10	0.61
3	37.01	49.29	1.70	0.60	0.11	8.82	1.76	0.71
4	37.47	48.23	2.17	0.68	0.04	8.62	2.11	0.67
5	36.64	48.29	2.74	0.67	0.15	8.87	1.90	0.74
6	34.89	48.60	4.00	0.68	0.05	8.68	1.95	1.15
7	27.99	38.90	23.09	0.44	0.31	6.55	2.04	0.67
Mean	35.61	47.13	5.49	0.60	0.12	8.30	2.01	0.75
Standard deviation	3.53	3.65	7.80	0.09	0.09	0.90	0.15	0.18
	Thin elongated particles – Blue triangles							
8	45.57	36.00	13.17	0.29	0.07	2.58	0.73	1.59
9	38.26	35.09	16.34	0.65	0.03	7.26	0.63	1.75
10	42.71	31.59	17.44	0.56	0.02	5.33	0.46	1.90
11	43.49	29.77	16.90	0.59	0.08	4.62	0.40	4.14
12	35.60	26.74	34.11	0.25	0.00	1.93	0.51	0.84
Mean	41.13	31.84	19.59	0.47	0.04	4.34	0.54	2.04
Standard deviation	4.08	3.81	8.28	0.18	0.03	2.15	0.13	1.24
	Fine material associated to the elongated particles – Red triangles							
13	41.49	29.47	25.79	0.28	0.08	1.24	0.42	1.23
14	43.68	29.17	24.26	0.19	0.00	1.00	0.23	1.48
15	48.26	24.65	23.58	0.17	0.03	1.29	0.17	1.86
16	41.42	29.37	25.45	0.34	0.06	1.82	0.29	1.24
Mean	43.71	28.16	24.77	0.24	0.04	1.34	0.28	1.45
Standard deviation	3.21	2.35	1.03	0.08	0.03	0.35	0.11	0.29
	Groundmass of surrounding submillimetric granular aggregates – Red circles							
17	53.90	11.55	30.30	0.06	0.18	0.05	0.15	3.82
18	52.46	12.33	32.94	0.00	0.16	0.07	0.00	2.04
19	47.02	12.10	38.60	0.09	0.07	0.24	0.06	1.81
20	65.54	9.82	22.31	0.01	0.00	0.09	0.06	2.16
Mean	54.73	11.45	31.04	0.04	0.10	0.11	0.07	2.46
Standard deviation	7.79	1.13	6.77	0.04	0.08	0.09	0.06	0.92
